# A Comparative Study on the Roll-to-Roll Processing of a Silicate–Polyvinyl Alcohol Composite Barrier Lacquer Using Slot-Die and Reverse Gravure Coating Techniques

**DOI:** 10.3390/polym15132761

**Published:** 2023-06-21

**Authors:** Stefan Schiessl, Esra Kucukpinar, Noémie Rivollier, Horst-Christian Langowski, Peter Eisner

**Affiliations:** 1 TUM School of Life Sciences Weihenstephan, Technical University of Munich, Alte Akademie 8, 85354 Freising, Germany; 2 Fraunhofer Institute for Process Engineering and Packaging IVV, Materials Development Giggenhauser Strasse 35, 85354 Freising, Germany; 3 Centre Technique Industriel de la Plasturgie (CT-IPC), 2 Rue Pierre et Marie Curie, 01100 Bellignat, France; 4 Institut für Geologische Wissenschaften, Freie Universität Berlin, Kaiserswerther Str. 16, 14195 Berlin, Germany; 5 Steinbeis-Hochschule, System- und Bioverfahrenstechnik, Ernst-Augustin-Straße 15, 12489 Berlin, Germany

**Keywords:** barrier lacquer, reverse gravure, oxygen permeability, montmorillonite, slot-die, polyvinyl alcohol, flexible packaging

## Abstract

The integration of platelet-shaped montmorillonite particles to improve the oxygen barrier of polyvinyl-alcohol-based barrier layers is state-of-the-art, but research on roll-to-roll coatings of such composite barrier lacquers has not been widely published. In this study, two different coating techniques, slot-die and reverse gravure, were used on a roll-to-roll scale to apply barrier lacquers comprising polyvinyl alcohol and montmorillonite. The lacquers were analyzed regarding viscosity at certain shear rates and surface energy and the dried coating layers regarding oxygen barrier, surface morphology, and particle orientation. Low permeability coefficients delivering a high oxygen barrier of 0.14 and 0.12 $\frac{{\mathrm{cm}}^{3}\;\left(\mathrm{STP}\right)\;1\;\mu m}{m^{2}\;d\;\mathrm{bar}\;}$ were achieved for the coating layers with slot-die and reverse gravure coating, respectively. It turned out that the properties of the barrier lacquer need to be adjusted to the coating technique to achieve high oxygen barrier performance. By tailoring the barrier lacquer formulation, the orientation of the platelet-shaped montmorillonite particles can be achieved using both techniques. A low solid content of down to 3 wt% is preferable for the premetered slot-die coating, because it results in low agglomeration quantity in the coating layer. A high solid content of up to 9 wt% is preferable for the self-metered reverse gravure coating to assure a homogeneously coated layer.

## 1. Introduction

Polyvinyl alcohol (PVA) is a widely applied and commonly used layer in flexible films intended for food packaging. It is chosen because it provides barrier properties against oxygen, biocompatibility, and biodegradability, as well as nontoxicity and easy film formation [1]. However, the availability of essential raw materials for production of PVA is limited [2]. The availability issue mainly concerns acetic acid, which is a precursor of vinyl acetate [3]. Of course, the global supply shortage causes prices to increase, e.g., in Europe by more than 100% from 2021 to 2022 [4,5]. Therefore, there is a strong interest in replacing PVA [6], producing it from biomass [7], reducing its amount [8] in gas barrier films, or reducing its content in packaging film in general.

In order to prevent the gas barrier deterioration of the packaging film due to the reduction in PVA content, nanoparticles can be added to keep the barrier performance at the same level despite reduced coating layer thickness. Numerous investigations have demonstrated that the barrier performance of PVA coatings can be improved by the addition of platelet-shaped silicate particles such as montmorillonite (MMT) [9,10,11,12,13,14]. These publications are restricted to sheet coatings at the lab scale. The transition between lab-scale and roll-to-roll scale applications is as challenging as the development of the coating material itself [15]. The limiting factors include (among others) the coating technique, the coating speed, the drying conditions, and the rheological properties of the lacquer [16,17]. Only a few publications have discussed the roll-to-roll coating of such silicate-based composite materials. Koppolu et al. [18] reported the roll-to-roll coating of microfibrillated cellulose or cellulose nanocrystals with the slot-die coating technique. In order to achieve an effective barrier using such cellulose-based coatings, they can be alternately coated with MMT dispersions to form a layer-by-layer structure, but this is then offset by the inefficiency of multiple coatings [19]. One potential layer-by-layer approach was also reported by Ben Dhieb et al. [11] for PVA and MMT dispersions, where blade coating was introduced as alternative coating technique to dip coating, but it was not performed on a roll-to-roll line. They achieved a low permeability coefficient of 0.2 $\frac{{\mathrm{cm}}^{3}\;\left(\mathrm{STP}\right)\;1\;\mu m}{m^{2}\;d\;\mathrm{bar}\;}$ and delivered a high oxygen barrier for 30 alternating layers coated with a doctor blade. Another feasible approach was introduced by LaChance et al. [20]. They demonstrated a one-time doctor-blade coating of a nanocomposite lacquer, also comprising PVA and MMT with an oxygen permeability of 2.1 $\frac{{\mathrm{cm}}^{3}\;\left(\mathrm{STP}\right)\;1\;\mu m}{m^{2}\;d\;\mathrm{bar}\;}$.

One possible field of application for these barrier lacquers is food packaging. Therefore, the barrier performance of the coating layer is compared with common barrier concepts in this application field, e.g., AlO${}_{x}$- or SiO${}_{x}$-deposited films or films with metallization [21,22]. Polypropylene films can reach permeation rates of down to 0.3 $\frac{{\mathrm{cm}}^{3}\;\left(\mathrm{STP}\right)}{m^{2}\;d\;\mathrm{bar}\;}$ with SiO${}_{x}$ deposition [23], down to 0.7 $\frac{{\mathrm{cm}}^{3}\;\left(\mathrm{STP}\right)}{m^{2}\;d\;\mathrm{bar}\;}$ with AlO${}_{x}$ deposition [24], and down to 0.5 $\frac{{\mathrm{cm}}^{3}\;\left(\mathrm{STP}\right)}{m^{2}\;d\;\mathrm{bar}\;}$ with metallization with aluminum [25]. Many of these barrier materials are used in laminate structures. Although packaging materials of only 12 to 20 μm are required to be vacuum-coated, the final packaging products may well be considerably thicker as they end up as a laminate. To ensure ease of handling for downstream processes and thus maintain the best barrier performance, most vacuum-coated barrier films are laminated [26].

As an alternative to these coatings in vacuum, this study presents a barrier lacquer that can be coated at atmospheric pressure. For this purpose, the aim is to investigate the roll-to-roll coating of a composite barrier lacquer comprising PVA and MMT using the slot-die or reverse gravure technique for the first time in detail to the author’s knowledge. The question to answer is how these two application techniques affect the layer homogeneity, surface morphology, agglomerate formation, and gas barrier performance. Therefore, a lacquer previously developed by Schiessl et al. [14] was prepared at three different solid contents of 3, 6, and 9 wt% in order to modify viscosities. The prepared lacquers were applied by means of a semiautomated roll-to-roll coating machine using two different coating techniques: slot-die and reverse gravure. The effect of the shear rate on the coating material was taken into consideration in addition to the lacquer viscosity and solid content effects. As a result, two different ceramic gravure rolls, with quadrangular and trihelical geometry, and two different shim thicknesses (gap openings) during the slot-die coating were used for each lacquer. This approach enabled investigations of various coating process windows with different parameter sets of lacquer viscosity and shear rate for both of the application techniques presented. The flexible films comprising a dried barrier layer were characterized in terms of surface roughness, orientation of MMT particles, and oxygen permeability coefficients of the coating layers. Based on the present research, a parameter set leading to low permeability coefficients delivering a high oxygen barrier was generated for every coating technique.

## 2. Materials and Methods

### 2.1. Materials

The platelet-shaped MMT particles (CLOISITE Na${}^{+}$ from BYK Additives and Instruments, Wesel, Germany) were dispersed using a planetary ball mill (PULVERISETTE 6, FRITSCH, Idar-Oberstein, Germany) and mixed with PVA (Exceval AQ-4104 from Kuraray, Frankfurt, Germany) at a mixing ratio of 1:1 by weight as reported by Schiessl et al. [14]. In contrast to the latter procedure, mixing and heating of the barrier lacquers were performed in a heatable blender (Thermomix TM5 from Vorwerk, Wuppertal, Germany) with a capacity of 2 L. The total solid content $c_{\mathrm{solid}}$ of the barrier lacquers was primarily adjusted to 9  wt% and subsequently diluted to 6 and 3 wt% with demineralized water using the same blender. The samples of the liquid barrier lacquers (BL) were labeled as BL-3, BL-6, and BL-9. A lower limit of 3 wt% solid content was chosen to provide a stable coating layer at the same drying conditions as the other coatings. The upper limit was set to 9 wt%, because, given their excessive viscosity, it was not possible to homogeneously coat lacquers having a higher solid content.

Biaxially oriented polypropylene (BoPP) was used as a substrate on rolls with a width of 300 mm and several hundred meters in length. BoPP was purchased as transparent, nonsealable film with a thickness of 30 μm (TNS from Taghleef Industries, San Giorgio, Italy). The BoPP film had a unit weight of 27.3 $\frac{g}{m^{2}}$, and in machine direction, a tensile strength of 150 MPa and an elongation at break of 140%.

### 2.2. Coating Processes

#### 2.2.1. Slot-Die Coating

**Figure 1:**
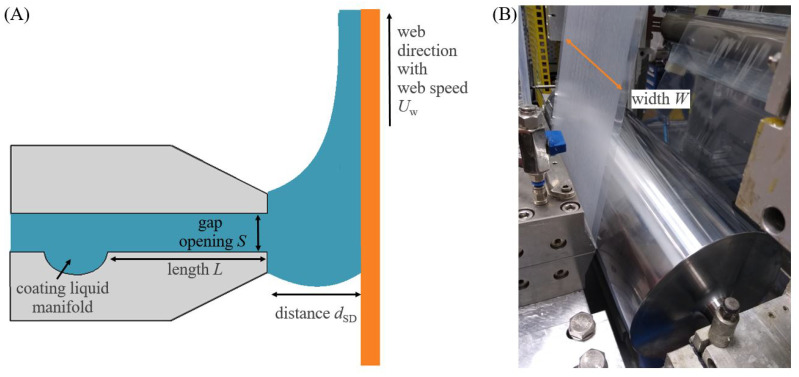
(**A**) Schematic drawing of the slot-die coating process illustrating the parameters of gap length *L*, shim thickness *S*, and distance from die to web $d_{\mathrm{SD}}$. (**B**) Picture of slot-die coating illustrating the coating width *W*.

The slot-die (SD) technique, shown in Figure 1, is a premetered process, i.e., the amount of liquid applied to the web per unit area is precisely adjusted by the volume flow [27]. The coatings were applied using a T-shaped manifold half circle slot-die (FMP Technology, Erlangen, Germany). The slot-die width *W* was 260 mm, and the gap length *L* in the slot-die was 45 mm. The shim thickness *S* was adjustable by using different shim plates from 100 to 500 μm. The wet coating layer thickness $t_{\mathrm{wet}}$ needed to achieve a defined dry layer thickness $t_{\mathrm{dry}}$ is calculated by

(1) $$t_{\mathrm{wet}}=\frac{t_{\mathrm{dry}}}{c_{\mathrm{solid}}}$$

The dry coating layer thickness was adjusted to 1.5 μm for all coatings using the slot-die, thus leading to the wet layer thicknesses listed in Table 1 for all parameter sets. A total of six different coating parameter sets, labeled as SD-500-9, SD-300-9, SD-300-6, SD-200-6, SD-200-3, and SD-100-3, were used to coat the BoPP. The volume flow $\dot{Q}$ dispensed by alternately pumping syringe pumps (Base 120 with two Nemesys S syringes, CETONI, Korbussen, Germany) required to achieve a wet layer thickness at a given web speed $U_{w}$ of 5 $\frac{m}{\min}$ is calculated by

(2) $$\dot{Q}=t_{\mathrm{wet}}\;\cdot \;U_{w}\;\cdot \;W$$

as listed in Table 1. Before reaching the slot-die, the barrier lacquer was pumped through a polymeric filter with a mesh size of 100 μm. Regarding the shear rates $\dot{\gamma }$ during the coating with slot-die, there are two important locations to consider, the shear rate within the die ${\dot{\gamma }}_{\mathrm{in}}$ and the shear rate directly before reaching the web ${\dot{\gamma }}_{\mathrm{SD}}$. A plane Couette flow develops in the slot-die, and the shear rate is calculated by

(3) $${\dot{\gamma }}_{\mathrm{in}}=\frac{6\;\cdot \;\dot{Q}}{W\;\cdot \;S^{2}}$$

according to Durst et al. [28]. The shear rate in the slot-die must be calculated to obtain the present viscosity η in the die, which in turn is needed to calculate the pressure loss Δp in the slot-die [29].

(4) $$\Delta p=\frac{12\;\cdot \;\eta \;\cdot \;L\;\cdot \;\dot{Q}}{S^{3}\;\cdot \;W}$$

A specific dynamic pressure in the slot-die is necessary for the coating liquid to be distributed homogeneously over the entire die [27]. The resulting pressure loss should be as low as possible for energy and safety reasons (bursting of hoses), and it was below a set value of 0.4 bar in any case, as shown in Table 1.

The slot-die was operated in bead mode for each coating, meaning that the distance $d_{\mathrm{SD}}$ between web and die was 2 to 5 times higher than $t_{\mathrm{wet}}$ [30]. Since the gap flow was already fully developed after a development length of less than 1 μm, the shear rate before touching the web was

(5) $${\dot{\gamma }}_{\mathrm{SD}}=\frac{U_{w}}{d_{\mathrm{SD}}}$$

when using the setup and coating fluids described [28,31,32]. The development length describes the length that is needed for a liquid to build up a constant flow without turbulence in a certain environment. Turbulent flow within the slot-die gap takes place for only several hundred nanometers after the liquid manifold. The subsequent flow can be described as fully developed.

#### 2.2.2. Reverse Gravure Coating

**Figure 2:**
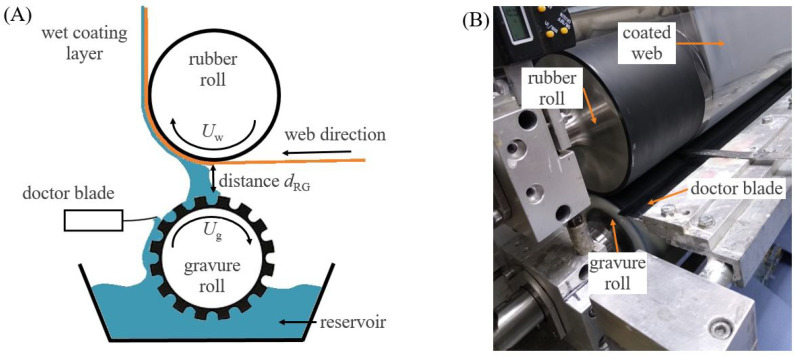
(**A**) Schematic drawing of reverse gravure coating process with delineated roll directions $U_{w}$ and $U_{g}$ representing the speed of substrate (web) and gravure roll, respectively. The distance between gravure roll and substrate is $d_{\mathrm{RG}}$ (adapted with permission from Shim [33], 2023, Elsevier). (**B**) Picture of reverse gravure coating process with quadrangular gravure roll.

The reverse gravure (RG) coating, as shown in Figure 2, was performed on two different gravure rolls. One of them was a ceramic gravure roll with quadrangular geometry (RG-q) with 65 μm in depth with a theoretical coating volume of 25 $\frac{\mathrm{mL}}{m^{2}}$ made by UNGRICHT (Moenchengladbach, Germany). The second was a ceramic gravure roll with a 45° trihelical geometry (RG-t) and 40 $\frac{\mathrm{lines}}{\mathrm{cm}}$, leading to a theoretical coating volume of 45 $\frac{\mathrm{mL}}{m^{2}}$ made by JWS (Sinsheim, Germany). In comparison with the slot-die coating technique, coating with reverse gravure is understood to be a self-metered process, which means that the wet layer thickness depends mainly on the gravure roll type but also on the solid content and flow behavior of the lacquer, the web speed, the surface energy of substrate and of the lacquer, and the interactions of these parameters with each other [27]. Barrier lacquers with a solid content of 3, 6 and 9 wt%, respectively, were coated using the two gravure rolls, leading to six different coating parameter sets with the sample codes RG-q-3, RG-q-6, RG-q-9, RG-t-3, RG-t-6, and RG-t-9. The coating width was 270 mm, and the web speed was similar to that of the slot-die coating 5 $\frac{m}{\min}$. The speed of the gravure roll $U_{g}$ was set to 7 $\frac{m}{\min}$ for all the parameter sets. Given that the development lengths for a fully developed flow are similar in both coating methods, the shear rate on the barrier lacquer before touching the web was calculated in a manner similar to the slot-die coating process. Therefore, a constant shear rate in both gaps, between die and web and between gravure roll and web $d_{\mathrm{RG}}$, is present [28,31].

(6) $${\dot{\gamma }}_{\mathrm{RG}}=\frac{U_{w}+U_{g}}{d_{\mathrm{RG}}}$$

#### 2.2.3. Roll-to-Roll Machine Adjustments and Curing

The slot-die and reverse gravure coating techniques are inherent to a semiautomated roll-to-roll coating machine. The BoPP substrate was placed in the unwinding station and passes consecutively through the corona pretreatment station, the coating station, the curing station, and the rewinding station, consecutively. During this process, the web tension at the unwinding station was always a little lower than the tension adjusted at the rewinding station due to the friction losses at several intervening rolls. The roll-to-roll coating line used was designed such that the coated side of the substrate never makes contact with a roll. The web tension for the BoPP substrate was at the rewinding station 30 N and at the unwinding station 20 N.

The substrate was physically pretreated using a corona discharge station (CLNE from SOFTAL electronic, Hamburg, Germany) to ensure a homogeneous wetting with lacquer. The plasma dose was adjusted to 4.8 $\frac{\mathrm{kg}}{m^{2}}$ for the pretreatment of BoPP in order to achieve a surface energy sufficient for wetting with aqueous barrier lacquers. In this context, “sufficient” means that the surface energy was measured to be more than 36 $\frac{\mathrm{mJ}}{m^{2}}$ by means of test inks (Teststifte PINK, arcotest, Moensheim, Germany, according to DIN ISO 8296 [34]). This is required for wetting with water-based coating liquids [35]. The curing was performed with a convection oven directly after the coating station. The curing conditions were 60 ${}^{\circ }C$, applied for 50 s. At least 50 m of film length was coated with every parameter set.

### 2.3. Methods

#### 2.3.1. Thickness Measurement

The thickness of the coating layer was measured using a precision thickness gauge (Hanatek FT3 from Rhopoint Instruments, Beyhill on Sea, UK). The resolution was 0.1 μm, the thickness was measured at five random spots on the coated sample, and an average value was then reported.

#### 2.3.2. Oxygen Permeability

The oxygen permeation rates of the coated films were measured according to the DIN 53380-3 standard with the oxygen-specific carrier gas method [36]. The measurements were performed using an OX-TRAN 2/21 OTR Analyzer (AMETEK MOCON, Minneapolis, MN, USA) at 23 °C and 50 %RH. If the respective permeation rate was constant for at least 10 h, then a steady state was assumed to have been achieved, and the measurement was then stopped. Duplicate determination was performed for all of the coatings, and the values were reported at standard deviation. After measuring the oxygen permeation rate of the coated film, the permeability coefficient of the coating layer was calculated using the measured total permeation rate of the coated substrate and the permeation rate of uncoated BoPP, which is 1250 $\frac{{\mathrm{cm}}^{3}\;\left(\mathrm{STP}\right)}{m^{2}\;d\;\mathrm{bar}\;}$. In this context, the value of the permeability coefficient is expressed as normalized to a material thickness of 1 μm, as described by Langowski [37].

#### 2.3.3. Surface Energy Measurement

The surface energy of the substrate–air interface was determined according to DIN EN ISO 19404-2 [38] via the sessile drop method with a DSA 100 from Kruess (Hamburg, Germany). For this purpose, at least five drops of three different liquids, water, ethylene glycol, and diiodomethane, were placed on the surface, and the contact angle was measured at equilibrium. At least two liquids with known polar and dispersive energy components are needed to calculate the polar and dispersive components of the surface energy using the Owens–Wendt–Rabel–Kaelble (OWRK) evaluation method [39].

The surface energy of the lacquer–air interface was measured according to DIN EN ISO 1904-3 [40] via the pendent drop method using the same device.

#### 2.3.4. Distance Measurement during Coating Processes

The values for $d_{\mathrm{SD}}$ and $d_{\mathrm{RG}}$ were measured in a similar way. First, the slot-die or the gravure roll were brought in contact with the substrate. This position was set as the zero point. The distance was then increased stepwise until reaching the final position for the respective parameter set and measured with a high-precision sensor head, GT2-H12K, from Keyence (Neu-Isenburg, Germany). The resolution was 10 μm.

#### 2.3.5. Viscosity Measurement

The rheological measurements were performed using a modular compact rheometer 302 from Anton Paar (Graz, Austria) equipped with a cone–plate set-up with a 50 mm diameter, an angle of 0.995°, and a preadjusted gap size of 100 μm. Since shear force is permanently applied in one direction during both of the coating processes used in the present study, the viscosity measurements were performed with a rotating plate. The shear sweep measurements of the lacquers produced were performed at room temperature, i.e., 23 °C, from 10 to 10,000 $\frac{1}{s}\;$ with a logarithmically increasing step size limited to 30 measuring points.

#### 2.3.6. Roughness Measurement

The roughness was measured via direct mechanical contact over the surface with a Hommel Tester W55 from Hommelwerke (Villingen-Schwenningen, Germany). For each sample, 5 lines with a length of 4.5 mm were measured parallel (MD) and perpendicular (TD) to the coating direction, and the roughness is given as an arithmetic mean value ($R_{a}$) of these 5 measurements, respectively.

#### 2.3.7. Scanning Electron Microscopy (SEM)

The SEM images were captured with a JSM-7200F (JEOL, Akishima, Japan) at high vacuum ( $2\times 10^{-4}$ Pa). All samples were sputtered with a gold layer using a Hummer JR sputter coater (Technics, Alexandria VA, USA) in order to reduce electrical charging of the nonconductive polymer samples. A solid-state detector (SSD), which detected backscattered electrons, was used in combination with the JEOL-SEM software Ver. 7.1.0.9. To perform a cross-section image, the coated substrate was placed between copper tapes with electrically conductive adhesive, and cross-cuts were prepared by Ar${}^{+}$ beam milling with a cross-section polisher (JEOL, Akishima, Japan).

#### 2.3.8. Pole Figures

Pole figures were measured using a PANalytical MRD X-ray diffractometer (Almelo, The Netherlands) equipped with a Eulerian cradle employing two-axis scans along ϕ and χ. Measurements were performed at a fixed 2θ angle of 6°, corresponding to the 2θ angle position of the lattice plane (002) studied. To plot pole figures, the intensity distribution was measured along the ϕ axis from 0 to 360° at sample tilts χ from 0 to 85°, with an increment of 5° each.

## 3. Results and Discussion

### 3.1. Oxygen Permeability

The coating thicknesses required to normalize the oxygen transmission rate and obtain the oxygen permeability coefficient are shown in Table 2. As expected, the thickness values for the premetered slot-die coating technique were all close to the target of 1.5 μm.

The coating layer thicknesses for the self-metered reverse gravure technique were difficult to adjust because of the dependency of the wet layer thickness on various parameters, as described in Section 2.2.2. Indeed, the higher the solid content, the thicker the coating layer. This relationship was valid for both gravure roll geometries. The thickness of the coatings with the trihelical gravure roll is measured as 1.9 μm, 1.3 μm, and 0.5 μm for the solid contents of 9, 6 and 3 wt%, respectively. Generally, the coating thicknesses achieved with the quadrangular gravure roll were found to be lower due to the lower theoretical coating volume of 25 $\frac{\mathrm{mL}}{m^{2}}\;$ compared with the trihelical gravure roll with 45 $\frac{\mathrm{mL}}{m^{2}}\;$. The coating layer thickness for RG-q-9 was 1.0 μm. For the barrier lacquers with lower solid contents of 3 and 6 wt%, the thicknesses with quadrangular gravure roll were found to be 0.1 and 0.3 μm. This corresponds to a wet layer thickness of only 3.3 and 5.0 μm, respectively. There are limits below which a stable film can no longer be formed, which is then reflected in very poor barrier properties. It was not possible to transfer more material to the substrate surface using the quadrangular gravure roll at these low solid contents.

**Figure 3:**
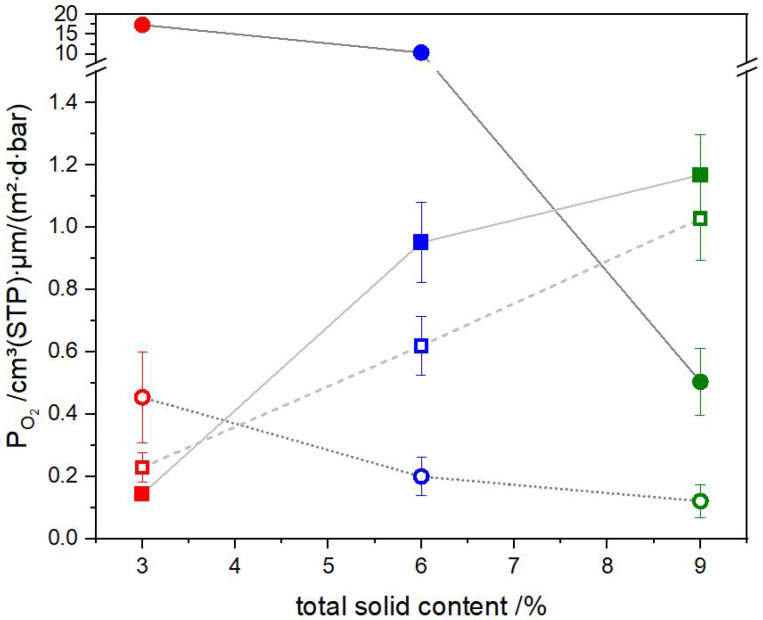
Oxygen permeability coefficients for the coating layers depending on the solid content of the barrier lacquer: The colors red, blue, and green represent the solid content of the barrier lacquers of 3, 6 and 9 wt%, respectively. The square symbols represent the slot-die coating, and the circle symbols represent the reverse gravure coating. A filled symbol represents the higher shear rate within a solid content, i.e., the quadrangular roll in the reverse gravure technique and the thin shims in the slot-die (300 μm for 9 wt%, 200 μm for 6 wt%, and 100 μm for 3 wt%). The empty symbol represents the lower shear rate within a solid content, i.e., the trihelical roll in the reverse gravure technique and the thick shims in the slot-die ( 500 μm for 9 wt%, 300 μm for 6 wt%, and 200 μm for 3 wt%).

Figure 3 shows the oxygen permeability coefficients of the barrier lacquers investigated. The previously reported permeability coefficient of this barrier lacquer obtained with a k-bar coating on BoPP sheets by Schiessl et al. [14] was 0.12 $\frac{{\mathrm{cm}}^{3}\;\left(\mathrm{STP}\right)\;1\;\mu m}{m^{2}\;d\;\mathrm{bar}\;}\;$ at a total solid content of 8 wt%. Similar values were achieved in this study. The lowest value with reverse gravure was 0.12 $\frac{{\mathrm{cm}}^{3}\;\left(\mathrm{STP}\right)\;1\;\mu m}{m^{2}\;d\;\mathrm{bar}\;}\;$ for RG-t-9, indicating the trihelical gravure roll and a solid content of 9 wt%. In the case of the slot-die coatings, the lowest value was 0.14 $\frac{{\mathrm{cm}}^{3}\;\left(\mathrm{STP}\right)\;1\;\mu m}{m^{2}\;d\;\mathrm{bar}\;}\;$ for SD-200-3, indicating a shim thickness of 200  μm and a solid content of 3  wt%.

The barrier performance of the slot-die-coated samples decreases by increasing the solid content, whereas the converse is true for the reverse-gravure-coated samples. Using slot-die coating, the permeability coefficients for BL-9 are similar, with values of 1.0 and 1.2 $\frac{{\mathrm{cm}}^{3}\left(\mathrm{STP}\right)\;1\;\mu m}{m^{2}\;d\;\mathrm{bar}\;}\;$, regardless of shear rate. Within the BL-6, the stronger sheared sample provides a permeability coefficient of 0.95 $\frac{{\mathrm{cm}}^{3}\left(\mathrm{STP}\right)\;1\;\mu m}{m^{2}\;d\;\mathrm{bar}\;}\;$ and the less sheared sample one of 0.62 $\frac{{\mathrm{cm}}^{3}\left(\mathrm{STP}\right)\;1\;\mu m}{m^{2}\;d\;\mathrm{bar}\;}\;$. For the BL-3, the low permeability coefficient of 0.23 $\frac{{\mathrm{cm}}^{3}\left(\mathrm{STP}\right)\;1\;\mu m}{m^{2}\;d\;\mathrm{bar}\;}\;$ is provided by the less sheared sample and the lowest value of 0.14 $\frac{{\mathrm{cm}}^{3}\left(\mathrm{STP}\right)\;1\;\mu m}{m^{2}\;d\;\mathrm{bar}\;}\;$ by the stronger sheared sample. Given that this does not represent a trend, it appears that the shear rate has little influence on the oxygen barrier performance of the coating layer. The main influencing factor during slot-die coating is the solid content and the properties accompanied with it.

The permeability coefficients for the samples coated with trihelical gravure roll, which are less sheared than the ones coated with quadrangular gravure roll, decrease from 0.45 to 0.12 $\frac{{\mathrm{cm}}^{3}\left(\mathrm{STP}\right)\;1\;\mu m}{m^{2}\;d\;\mathrm{bar}\;}\;$ as the solid content increases from 3 to 9 wt%. The lowest permeability coefficient, reached using the quadrangular gravure roll, was 0.50 $\frac{{\mathrm{cm}}^{3}\left(\;\mathrm{STP}\right)\;1\;\mu m}{m^{2}\;d\;\mathrm{bar}\;}\;$ at a solid content of 9  wt%. The barrier improvements reached with the quadrangular gravure roll at 3 and 6 wt% were much lower than those achieved using the trihelical gravure roll, which may have been due to the insufficient amount of material transferred, as shown in Table 2.

Samples RG-t-9, RG-t-6, RG-t-3, RG-q-9, SD-200-3, and SD-100-3, which were obtained using 6 different parameter sets, provided oxygen permeability coefficients below 0.5 $\frac{{\mathrm{cm}}^{3}\left(\mathrm{STP}\right)\;1\;\mu m}{m^{2}\;d\;\mathrm{bar}\;}\;$ on BoPP. The reasons for the differences among the 12 measured samples are discussed in the next sections.

### 3.2. Surface Energy of the Barrier Lacquers and Substrate

The surface energies of the barrier lacquers with various solid contents are shown in Table 3. In comparison with the surface energy of pure water with 72 $\frac{\mathrm{mJ}}{m^{2}}\;$[41], the surface energy of the barrier lacquers decreases to 62 $\frac{\mathrm{mJ}}{m^{2}}\;$ after mixing water with PVA and MMT. All three barrier lacquers exhibited nearly the same surface energy. It was shown by Chen et al. [42] that an increasing amount of MMT in an aqueous dispersion leads to an increase in surface energy. On the other hand, Nagarkar and Patel [43] found out that an increasing amount of PVA in an aqueous dispersion leads to a decrease in surface energy. The variation in total solid content in this study did not affect the surface energy. This outcome may have been due to the combination of PVA and MMT effects in the formulation used having counteracted one another.

One of the major requirements for a strong bond between a barrier lacquer and a polymeric substrate is that the liquid lacquer completely covers the substrate surface during the coating process, i.e., wettability [44]. If a lacquer does not wet, defects such as islands will appear during the coating process, even before curing of the lacquer, which leads to degradation of the functional properties. A high level of wettability can be achieved if the total surface energy of the coating formulation is lower than the total surface energy of the substrate [45]. The surface energy of untreated BoPP with 24 $\frac{\mathrm{mJ}}{m^{2}}\;$ was found to be too low in comparison with the surface energy of the barrier lacquers. After corona pretreatment, however, the surface energy increased to 37 $\frac{\mathrm{mJ}}{m^{2}}\;$, mainly due to the significant increase in the polar component by 6.8 $\frac{\mathrm{mJ}}{m^{2}}\;$. This increase was in agreement with values previously reported by Lindner et al. [46] and Aydemir et al. [35]. Although the total surface energy of BoPP after the corona pretreatment was still lower than the surface energy of the barrier lacquers, it was possible to spread the barrier lacquers spontaneously on the substrate surface by means of slot-die and reverse gravure coating. It was discovered that the viscosity of the barrier lacquer formulations, the wet coating layer thickness, and the coating parameters have additional influences on the layer coating performance, as was demonstrated by previous research [35,45,47,48].

The foregoing findings enable conclusions to be made about the oxygen barrier performance of the barrier lacquers (Section 3.1). If the BL-3 is, e.g., coated at a certain wet layer thickness, in the case of SD-200-3 with 50 μm, then the substrate will be wetted completely. Otherwise, a barrier value of 0.14 $\frac{{\mathrm{cm}}^{3}\left(\mathrm{STP}\right)\;1\;\mu m}{m^{2}\;d\;\mathrm{bar}\;}\;$ for slot-die coating would not have been measured. However, if the BL-3 is coated too thinly, as was the case for quadrangular gravure roll with only 3.3 μm, then a few uncoated areas may remain. This would explain why the permeability coefficient, which is independent of the coating layer thickness, was much higher for the same lacquer in sample RG-q-3 comprising 17 $\frac{{\mathrm{cm}}^{3}\left(\mathrm{STP}\right)\;1\;\mu m}{m^{2}\;d\;\mathrm{bar}\;}\;$. Therefore, the explanation is that the resulting force from the surface energy mismatch needed to form an island is higher than the gravitational force that holds the lacquer in place. Barrier lacquer BL-6, which had a similarly thin coating of 5.0 μm as BL-3 but a higher viscosity (see Section 3.4), also provided a high permeability coefficient of 10 $\frac{{\mathrm{cm}}^{3}\left(\mathrm{STP}\right)\;1\;\mu m}{m^{2}\;d\;\mathrm{bar}\;}\;$ for the RG-q-6 sample. This result demonstrates that the combination of viscosity, wet coating layer thickness, and surface energy needs to be sufficient to wet the substrate homogeneously.

### 3.3. Shear Rates during Coating Process

**Figure 4:**
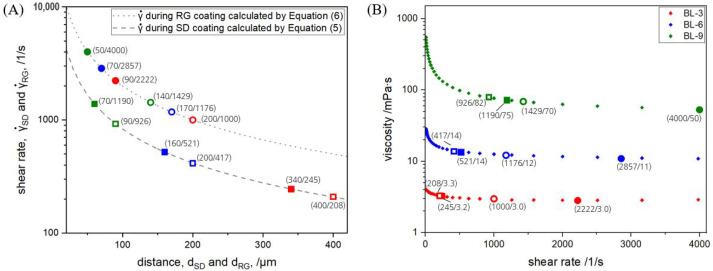
(**A**) The calculated shear rate during slot-die and reverse gravure coating plotted as a function of the measured distance between the slot-die and substrate $d_{\mathrm{SD}}$ or between gravure roll and substrate $d_{\mathrm{RG}}$, respectively. (**B**) The viscosity of the barrier lacquers depending on the shear rate. The colors red, blue, and green represent the solid content of the barrier lacquers of 3, 6 and 9wt%, respectively. The square symbol represents the slot-die coating and the circle symbol the reverse gravure coating. A filled symbol represents the higher shear rate within one solid content and the empty symbol the lower shear rate. The highlighted dots show the exact shear rates that the lacquers were subjected to during coating with the corresponding numerical values in (x/y) form.

Figure 4A illustrates the shear rates during the two coating processes as a function of the distances between slot-die and web or between gravure roll and web. The shear rates to which the lacquer is subjected during both coating techniques are calculated according to Equations (5) and (6), respectively.

The difference between reverse gravure and slot-die coating in terms of shear rate is best described using an example, i.e., BL-9. Sample BL-9 was sheared at 1190 and 926$\frac{1}{s}\;$ and distances of 70 and 90 μm, respectively, during the slot-die process but at 4000 and 1429$\frac{1}{s}\;$ and distances of 50 and 140 μm during the reverse gravure process. Within the slot-die coating, BL-9 was sheared at 925 $\frac{1}{s}\;$ for a shim of 300 μm but at 1190 $\frac{1}{s}\;$ for a shim of 500 μm. Within the parameter set of the thicker shims, as indicated by the filled symbols in Figure 4A, the shear rate decreased from 1190 to 245$\frac{1}{s}\;$ due to the distance increasing from 70 to 340 μm for solid content decreasing from 9 to 3 wt%. The shear rates during the slot-die process were lower than those occurring during the reverse gravure process. Within the slot-die process, it was found that the shear rate decreased with a decreasing shim thickness (increasing distance between die and web) within one solid content or with a decreasing solid content. The distances, $d_{\mathrm{SD}}$ and $d_{\mathrm{RG}}$, were adjusted in a way to provide the best coating picture and were measured for every coating parameter set. In this context, a good coating picture means a wet coating layer without any ribbings, striations, voids, or other inhomogeneities. The highest possible distance providing a good coating picture is preferred for the barrier lacquers, as was demonstrated by Creel et al. [49] for polymer electrolyte fuel cell cathode ink. Bhamidipati et al. [50] showed that the potential for air entrainment is higher for higher viscosity and that it can be reduced by reducing the distance from die to web. This finding was applied within this study, since an air entrainment would lead to the aforementioned inhomogeneity and needs to be avoided. Even if entrained bubbles in the liquid coating layer are not visible during coating, they still result in an inhomogeneous appearance after drying. This is due to the fact that the thermal expansion coefficient of gases is three times higher than that of solids, and the encapsulated bubbles almost burst open during drying [51]. The viscosity of BL-9 was higher than that of BL-3 (see Section 3.4), for which reason the distance was lower for BL-9.

In the case of reverse gravure coatings, the distance for the quadrangular gravure roll extended from 50 to 90 μm and led to shear rates from 4000 to 2222 $\frac{1}{s}\;$ for decreasing solid content. The distance range for the trihelical gravure roll was from 140 to 200 μm, resulting in shear rates from 1429 to 1000 $\frac{1}{s}\;$. The distance increased with an increasing coating volume, which led to a lower shear rate. Within one roll geometry, the distance for the lacquer with higher solid content is lower because of two reasons. Firstly, it is similar to the slot-die coating, i.e., the higher the viscosity, the higher the risk of air entrainment with increasing distance. Secondly, since the surface energy of the lacquers was quite similar (see Table 3), the only varying parameter in the law of capillary rise is the density, and the density for lacquers with higher solid content was higher, so the capillary rise is lower [52].

### 3.4. Viscosity of Barrier Lacquers

Figure 4B shows the viscosity of the barrier lacquers prepared at three different solid contents as a function of shear rate. The shear rates applied on the barrier lacquers during the coating processes (Figure 4A) are marked on the diagram.

Sample BL-9 exhibited a shear-thinning behavior, as quantified by a viscosity reduction from 550 mPa s at a shear rate range of 10 $\frac{1}{s}\;$ to 50 mPa s at a shear rate of 4000 $\frac{1}{s}\;$. The viscosity reduction for BL-6 and BL-3 lasted from 28 to 11 mPa s and from 4.0 to 3.0 mPa s for the same shear rate range, respectively. At a shear rate of 4000 $\frac{1}{s}$, the viscosity increased from 3.0 to 50 mPa s for an increasing solid content from 3 to 9  wt%. In both coating techniques, the parameter sets used led to a shear rate, which was in turn accompanied by a viscosity that started to become approximately constant, or at least to a viscosity not in the area of strong exponential decay at low shear rates from 10 to 200 $\frac{1}{s}\;$.

All of the formulations provided a shear-thinning behavior similar to PVA solutions [53] and MMT dispersions [54]. Without any shear force, the PVA chains were oriented randomly in the lacquer, as were the MMT platelets. As soon as the lacquer is sheared, the chains and platelets start to orient, which results in a decrease in viscosity. This effect became increasingly pronounced with an increasing lacquer solid content, as described by, e.g., Xu et al. [55]. When the majority of chains and platelets were oriented, the viscosity no longer changed significantly. At this shear rate, when the viscosity became nearly constant, the barrier lacquers should be coated. As expected, the viscosity increased with an increasing solid content because fewer water molecules are mobile and more water molecules are part of hydration shells, either from PVOH or from MMT. For the single components of the barrier lacquers, PVA and MMT, it is also true that the viscosity increases with increasing solid content [56,57].

### 3.5. Roughness

**Figure 5:**
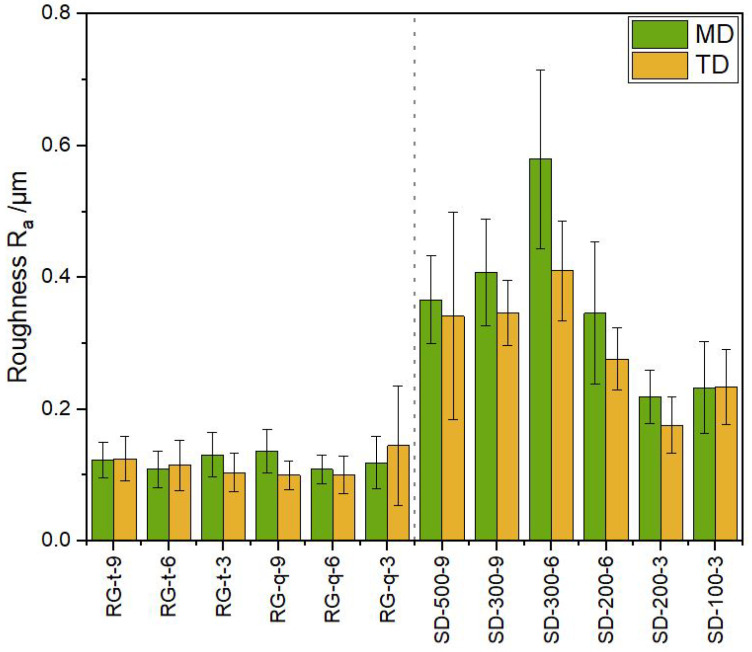
Arithmetic mean values for the roughness of the coated BoPP substrate measured parallel (MD) and perpendicular (TD) to the coating direction.

Figure 5 shows the value most commonly used to describe the roughness of coated surfaces (the arithmetic mean value $R_{a}$) for the coating parameter sets. The roughness in coating direction did not differ from the perpendicular direction in either coating technique.

Regarding reverse gravure coating, the roughness values were all within a range of 0.1 to 0.14 μm. In comparison, slot-die coated samples varied from 0.18 to 0.58 μm, with SD-200-3 being the smoothest at 0.18 μm and SD-300-6 being the roughest at 0.58 μm. Counterintuitively, the roughness values of the reverse-gravure-coated samples were all below the roughness values measured for the slot-die-coated samples. Since the roughness values were similar in the two different gravure geometries and all of the solid contents measured, it can be concluded that the gravure roll type had no direct influence on the roughness for the setups and barrier lacquers used. This outcome stands in contrast to the finding by Voigt et al. [58] for gravure coatings of similar viscous polythiophene but with a different gravure geometry and setup.

**Figure 6:**
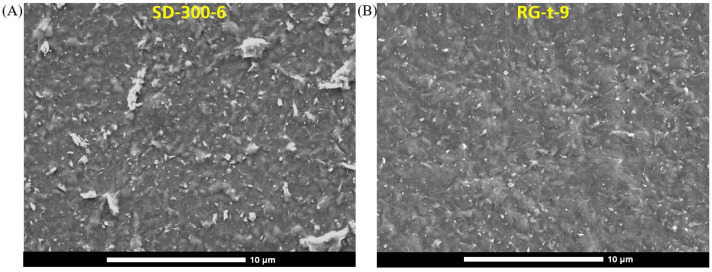
Top view SEM image of the coated substrate with a magnification of 5000: (**A**) SD-300-6 (**B**) RG-t-9.

The roughness is, among others things, determined by agglomerations on the surface of the coating. Figure 6 shows a comparison of the surface structure of two samples, SD-300-6 and RG-t-9. The surface of the sample SD-300-6 consists of several particles (whitish regions). These particles are MMT agglomerations. Since MMT contains heavier atoms in terms of atomic number compared with PVA, MMT appears brighter in the SEM image. Three reasons can be identified for why the potential for agglomerations during slot-die coating was higher than during reverse gravure coating.

Since the slot-die coating technique is premetered, any agglomeration formed during lacquer production which is smaller than the filter (mesh size 100 μm) ends up in the coating layer. Additionally, during the slot-die coating process, there are some zones that act as dead volume within the die, for example, in the coating liquid manifold or on the very edges of the die gap. Spontaneously formed agglomerations at these zones also enter the coating. Finally, the agglomerations formed during slot-die coating face lower shear rates within the range of 1190 to 208 $\frac{1}{s}\;$ than those applied during reverse gravure coating (Figure 4).

In the reverse gravure process, on the other hand, agglomerations formed during barrier lacquer production are not completely transferred to the substrate and also remain in the reservoir. Moreover, the agglomerations transferred by the gravure roll are actually sheared at a higher shear rate of 4000 to 1000 $\frac{1}{s}\;$ (Figure 4) and reduced in size before coating, or they are blocked via the doctor blade and returned to the reservoir. It was demonstrated earlier with regard to this barrier lacquer that a larger agglomeration quantity results in a lower barrier potential of the barrier lacquer [14], and this phenomenon was further confirmed by the roll-to-roll coatings using two coating techniques.

### 3.6. Orientation of Montmorillonite Particles in Dry Coating Layer

The commonly held opinion is that the orientation of the platelet-shaped MMT particles has a decisive influence on barrier performance [59]. Therefore, the orientation of the MMT particles in the roll-to-roll-coated samples was investigated via two methods: quantitatively with a pole figure measurement of the (002) lattice plane of MMT and qualitatively with a cross-section SEM image.

**Figure 7:**
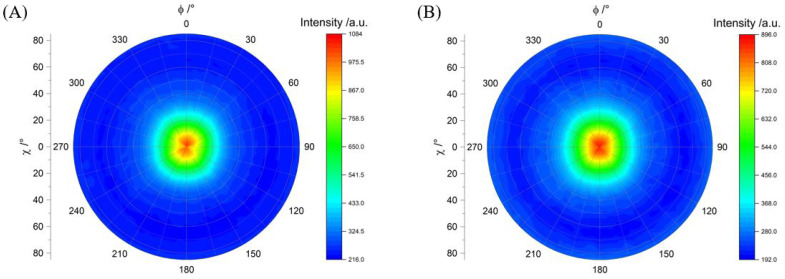
Pole figures of the (002) lattice plane of MMT determined for barrier lacquer coating on (**A**) SD-200-3 and (**B**) RG-t-9.

Figure 7 shows two pole figures of the (002) lattice plane, measured for a representative slot-die- and reverse-gravure-coated sample. Samples SD-200-3 and RG-t-9 were chosen by virtue of their low permeability coefficient, thereby delivering high oxygen barrier performance (see Section 3.1). The (002) lattice plane describes the parallel plane of an MMT platelet, where the reflection takes place on the next parallel plate. Hence, this lattice plane is used to observe the swelling and separation of the platelets, as well as their orientation [60]. Both pole figures exhibited a high intensity for χ angles from −10 to 10${}^{\circ }$, as indicated by the red coloring in the center, which corresponds to the (002) tilt angle to the substrate surface. This result revealed that the majority of the particles were oriented more or less parallel to the surface, with a maximum tilt of 10${}^{{}^{\circ}}$. Both samples exhibit a diffraction halo for χ from 10 to 30${}^{{}^{\circ}}$, as indicated by the color range from yellow to bright blue. This implies that a minority of MMT platelets provides larger tilts than 10${}^{{}^{\circ}}$. Based on the pole figure results, it appears that the coating techniques investigated had similar influence on the orientation, since the MMT orientation was comparable. The shear was strong enough to align the particles during both coating processes. On this basis, it can be concluded that complete wetting without defects and a lowest possible amount of agglomerations is required for a high barrier. This is the most reliably achieved for this lacquer in the reverse gravure process, as long as the minimum amount of lacquer is transferred (see Section 3.1).

**Figure 8:**
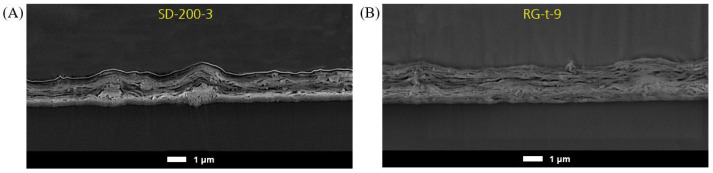
SEM image of cross-sections prepared by Ar polishing with a magnification of 15,000: (**A**) SD-200-3 and (**B**) RG-t-9.

The SEM cross-section images of the same sample pair (see Figure 8) reveal that the MMT particles are oriented horizontally with respect to the substrate surface, because a dominating direction of the bright lines is recognizable from left to right, i.e., parallel to the surface. However, regions having certain tilt angles are also present, especially around agglomerations, e.g., in SD-200-3. It is important to keep in mind that a cross-section image only represents a small partial section of the whole sample. Therefore, the quantitative measurement with pole figure is more reliable for orientation investigation, because an average of a larger area is evaluated. The cross-section images confirm the findings described in Section 3.5, i.e., that the number of agglomerations is higher in slot-die-coated samples than in reverse-gravure-coated samples. Additionally, the cross-section image demonstrates that the agglomerations are not necessarily located on the surface but also within the layer.

During slot-die coating, the lowest permeation coefficient of 0.14 $\frac{{\mathrm{cm}}^{3}\left(\mathrm{STP}\right)\;1\;\mu m}{m^{2}\;d\;\mathrm{bar}\;}\;$ was obtained for the lacquer with the lowest solid content, BL-3. This lacquer also contained the lowest number of agglomerations, as shown by the roughness measurement in Section 3.5. The higher the solid content, the higher the potential for an agglomeration to build up during lacquer production or within the dead volumes during coating process. An agglomeration disturbs the parallel orientation of platelets to the surface, and the platelets that are agglomerated no longer contribute to the tortuous path effect anymore. Therefore, a low solid content is preferable during the slot-die process in order to minimize the number of agglomerations.

Given that the reverse gravure process is self-metered, another parameter becomes important, i.e., the layer thickness. As shown in Section 3.1, a specific layer thickness at which a completely closed layer is attained is required in order to achieve high oxygen barrier performance. If this layer thickness is reached (e.g., 0.5 μm in the case of RG-t-3), all of the permeability coefficients obtained are below 0.50 $\frac{{\mathrm{cm}}^{3}\left(\mathrm{STP}\right)\;1\;\mu m}{m^{2}\;d\;\mathrm{bar}\;}\;$, regardless of the shear rate. The reverse gravure coating process does not allow big agglomerations to enter the wet coating layer at all, but the agglomerations stay at the reservoir, are blocked by the doctor blade, or are reduced in size. The main factor influencing reverse gravure coating is the (specific) layer thickness, in combination with the viscosity of the lacquer. A lacquer having a high solid content and high viscosity is preferable for the reverse gravure process with the trihelical or quadrangular roll used.

## 4. Conclusions

This study provides a comparison of two coating techniques, reverse gravure and slot-die, using a roll-to-roll coating line. The focus is on the effecting parameters of the oxygen barrier performance of silicate composite barrier lacquers. The main factors investigated included the effect of the solid content, the reverse gravure geometry, and the shim thickness during the slot-die coating process. The permeability coefficients obtained using twelve different parameter sets were compared with each other.

The viscosity measurements demonstrated that the barrier lacquers comprising PVA and MMT provided shear-thinning behavior. The rheological investigation also showed that the viscosity of the lacquers increases with an increasing solid content. The solid content, however, has no influence on the surface energy of the lacquers. All barrier lacquers are measured to be similar with a value of 62 $\frac{\mathrm{mJ}}{m^{2}}\;$. The BoPP substrate was pretreated with corona discharge to be wettable for water-based barrier lacquers. A parameter set was obtained, which enables the achievement of a high oxygen barrier with a low permeability coefficients of 0.12 and 0.14 $\frac{{\mathrm{cm}}^{3}\left(\mathrm{STP}\right)\;1\;\mu m}{m^{2}\;d\;\mathrm{bar}\;}\;$ for both coating processes, reverse gravure and slot-die, respectively. While for the slot-die technique it was shown that low viscous lacquers lead to a high barrier, the opposite was shown for the reverse gravure process.

It was found that the amount of agglomeration in the cured barrier layer, in addition to a defect free coating, has the greatest effect on the barrier. The reverse gravure technique is better suited to reproducibly form a coating layer that has a smooth surface (Figure 5), contains few agglomerations (Figure 6) and, if it is ensured that the dry layer thickness is above 0.5 μm (Table 2), also shows excellent barrier properties (Figure 3). The agglomeration amount during slot-die coating is the lowest when the solid content is 3 wt%, because fewer agglomerations develop during the coating process.

In conclusion, the present research suggests parameter sets for slot-die and reverse gravure coating of a silicate-based composite comprising PVA and MMT. In addition, the findings regarding solid content, viscosity, shear rate, and roughness can also be adopted for other composite materials or coating fluids. One important finding hereby is that it is not simply possible to use the coating formulations of the laboratory scale one by one, but the coating formulation must rather be adapted to suit the roll-to-roll coating process in terms of its properties, e.g., viscosity and solid content. More specifically, for the lacquer comprising MMT and PVA, a high viscosity (70 mPa s) and high solid content (9 wt%) is preferable for the reverse gravure process, whereas a lower viscosity (3.2 mPa s) and a filtered lacquer with a low agglomeration amount and low solid content (3 wt%) is preferable for the slot-die coating technique.

In a future application, these BoPP films after coating with such a silicate composite barrier lacquer would be laminated to a PP sealing film. During the recycling process, these laminates are then shredded, easily separated, and can be recovered by type after the washing process due to the solubility of the lacquer in water.

## Figures and Tables

**Table 1:** Parameter sets used for coatings with slot-die (*S*: shim thickness, $c_{\mathrm{solid}}$: total solid content, $t_{\mathrm{wet}}$: wet layer thickness, $\dot{Q}$: volume flow, ${\dot{\gamma }}_{\mathrm{in}}$: shear rate, η: viscosity, and Δp: pressure loss).

	*S*	$c_{\mathrm{solid}}$	$t_{\mathrm{wet}}$	$\dot{Q}$	${\dot{\gamma }}_{\mathrm{in}}$	η	Δp
**Sample Code**	μ **m**	**wt%**	μ **m**	$\frac{\mathrm{mL}}{\min}\;$	$\frac{1}{s}\;$	**mPa s**	**bar**
SD-500-9	500	9	17	21.7	33.3	336	0.020
SD-300-9	300	9	17	21.7	92.6	201	0.056
SD-300-6	300	6	25	32.5	139	16.5	0.007
SD-200-6	200	6	25	32.5	313	14.7	0.021
SD-200-3	200	3	50	65.0	625	2.99	0.008
SD-100-3	100	3	50	65.0	2500	2.83	0.064

**Table 2:** Dry coating layer thickness measured for the various coating techniques and parameter sets. These values are used to normalize the permeation rate to calculate the permeability coefficients.

Self-Metered Reverse Gravure Coating		Premetered Slot-Die Coating	
	μ **m**		μ **m**
RG-t-9	1.90.1	SD-500-9	1.60.1
RG-t-6	1.30.1	SD-300-9	1.60.2
RG-t-3	0.50.2	SD-300-6	1.60.1
RG-q-9	1.00.1	SD-200-6	1.50.1
RG-q-6	0.30.2	SD-200-3	1.50.1
RG-q-3	0.10.1	SD-100-3	1.40.1

**Table 3:** Surface energy of BoPP before and after corona pretreatment measured using the sessile drop method giving the corresponding dispersive and polar components and surface energy measured with the pendant drop method of barrier lacquers with solid contents of 3, 6, and 9 wt%. The subdivision in dispersive and polar components is not applicable (N/A) with the pendant drop method.

	Total ($\frac{\mathrm{mJ}}{m2}$)	Dispersive ($\frac{\mathrm{mJ}}{m2}$)	Polar ($\frac{\mathrm{mJ}}{m2}$)
BoPP (untreated)	24.10.2	24.00.2	0.080.01
BoPP (pretreated)	37.00.4	30.10.3	6.940.12
BL-3	61.70.2	N/A	N/A
BL-6	62.30.2	N/A	N/A
BL-9	61.50.7	N/A	N/A

## Data Availability

The data presented in this study are available on request from the corresponding author. The data are not publicly available for reasons of confidentiality in the aforementioned EU projects; see section “Funding”.
